# Floor Substrate Preferences of Chickens: A Meta-Analysis

**DOI:** 10.3389/fvets.2020.584162

**Published:** 2020-12-09

**Authors:** Valerie Monckton, Jennifer L. Ellis, Alexandra Harlander-Matauschek

**Affiliations:** Department of Animal Biosciences, University of Guelph, Guelph, ON, Canada

**Keywords:** dustbathing, foraging, bedding, litter, *Gallus gallus domesticus*, Galliformes, pecking

## Abstract

Environmental enrichment promotes sensory and motor stimulation for species-typical behaviors, which in turn enhance animal well-being. For farmed Galliformes, housing systems often limit enrichment to bedding and litter, that simultaneously act as material for dustbathing and foraging. Therefore, this meta-analysis sought to systematically review and synthesize the substrate preference test literature for Galliformes. Data based on the following four welfare-related behaviors were extracted for analysis: (1) dustbathing, (2) foraging, (3) pecking, and (4) time spent on a given substrate. Literature searches in CAB Direct, Web of Science, and Google Scholar yielded 239 articles, and hand searching yielded an additional five articles. Ten publications that used different chicken strains as test subjects, met the criteria to be included in the systematic review. The effects of bedding type, the number of days birds had access to tested substrates, enclosure area, and substrate area, on the examined behaviors were determined. We found that birds preferred dustbathing in sand and peat moss more than on any other substrates. The bedding type, size of the enclosure, and size of the substrate area affected the amount of time that birds spent on the tested substrates. When provided the choice between bedding materials, birds spent more time on sand or peat moss than on any other substrate or on no substrate. Notably, most studies did not report relevant physical or chemical characteristics of substrate that may influence birds' preferences, such as grain size, moisture content and the level of soiling. Focusing future studies on identifying substrate characteristics that influence preferences can lead to the discovery of new, practical, enriching beddings that can be easily implemented in housing systems for Galliformes.

## Introduction

Galliformes encompass the largest group of farmed land animals, with chickens alone numbering 23.7 billion in 2018 (1). While many factors affect Galliformes' welfare, bedding is often overlooked and chosen principally based on price and regional availability (2), although other factors such as labor requirements, wear and tear on equipment, cost, accessibility, sustainability, and manure handling and storage may impact bedding choice as well. Yet, while bedding may vary globally, many farmers in Canada and the United States use wood shavings (2, 3). Choice of bedding has been shown to impact many aspects of health and welfare, including the air the birds (and farmers) breathe and the incidence of disease (4–8). Moreover, bedding can act as environmental enrichment—by enhancing “animal welfare through sensory and motor stimulation, using structures and resources that permit the expression of species-typical behaviors and promote psychological well-being through physical exercise, manipulative activities, and cognitive challenges according to species-specific characteristics” (9). As a substrate for foraging and dustbathing, bedding can increase Galliformes' behavioral repertoire, and reduce frustrated behaviors. For example, select beddings can reduce the incidence of injurious feather pecking, a repetitive and undesirable behavior common in many breeds of farmed Galliformes (10–12).

Commercial farms commonly use bedding in cage-free systems to absorb moisture from drinkers and birds' excreta, while cage systems may provide bedding in small areas as enrichment for birds to dustbathe and forage in, or beneath cages to absorb moisture from fallen excreta. Whether its primary purpose is moisture absorption or enrichment, bedding is invariably used to both regulate the barn environment and provide environmental enrichment.

To serve both these purposes well, good bedding must first efficiently absorb moisture quickly and facilitate its rapid evaporation, preventing moisture buildup that increases the incidence of disease. Indeed, high bedding moisture has been linked to higher rates of contact dermatitis, respiratory disease, and keratoconjunctivitis (13, 14). Moreover, a study by Drake et al. (15) found that the use of bell drinkers (open water) were a risk factor for the development of feather pecking in laying hens, thereby suggesting that moist and caked litter may contribute to adverse health conditions that could spur undesirable behaviors. However, like feather pecking, wet litter substrate is also less loose (friable) and more prone to caking, which makes it an inferior substrate for dustbathing.

Galliformes are motivated to dustbathe (16, 17), and will even dustbathe without substrate present (sham dustbathing) (18). Behavioral elements of dustbathing including vertical wing shaking, bill raking and head rubbing, with the goal of incorporating substrate throughout Galliformes' plumage (19). In so doing, it allows birds to remove stale lipids from their feathers (16, 19, 20) and dislodge ectoparasites (19, 21). Furthermore, poor or absent dustbathing substrate can lead Galliformes to become more fearful, leading to higher incidences of feather pecking (10, 22).

A combination of pecking and scratching, foraging occupies about 40% of chickens' daylight hours (23–25). While foraging is used by wild Galliformes as an appetitive behavior to find food, Galliformes will forage even in the presence of readily available feed, suggesting that the behavior itself is motivating (26, 27). Consequently, Galliformes' frustrated motivation to forage is strongly related to severe, injurious feather pecking of conspecifics (28), while the provision of high quality pecking substrates is suggested to protect against feather pecking [(29), van Staaveren, et al., submitted] and reduces fearfulness (10), acting as an enrichment. Therefore, providing foraging substrate—one that is nutritive (30) and that requires a longer amount of time to search, manipulate, and consume (11, 28)—can also act as enrichment.

First conducted in the 1970s, preference tests investigate where and how animals spend their time when provided different, but similar resources. These results are then used to ascribe preference (31). The preferences demonstrated are then presumed to present conditions that benefit the animals' health and well-being. Many external and internal factors, including animals' previous experiences, the time of day, the animals' mental state, and the differences between the presented options, can impact the outcome of a preference test. It is also important to consider that animals may make decisions that do not align with their welfare, and that their preferences are often restricted by the choices that humans offer them (31, 32).

Therefore, this meta-analysis used floor substrate preference studies to assess the bedding material that provided the best enrichment based on where Galliformes spent time, dustbathed, foraged, and pecked. Moreover, it sought to verify claims that peat and sand were preferred dustbathing substrates, and that Galliformes do not have a preference for foraging substrates (33, 34). Although bedding type is the focus of this review, many studies included did not distinguish between litter and bedding. Therefore, this review uses the term “substrate” to refer to both bedding (fresh material) and litter (bedding mixed with excreta, feathers and waste feed).

## Materials and Methods

### Database

Three primary groups of search terms were used to amass literature. These terms were (1) bedding, litter, or substrate, (2) Galliformes, poultry, fowl, chicken, turkey, or galliform, and (3) preference, motivation, behavior, behavior, forage, foraging, dustbathing, or dust bathing. The search terms were used to hand-search for literature, and to search the following databases: Web of Science (Thomson Reuters Science, New York, NY), CAB Direct (CAB International, Wallingford, UK) and Google Scholar (specified to Animal Welfare and Applied Animal Behavior journals). The search was conducted between January and March 2020, and there were no time period exclusion criteria. The search resulted in 239 related references. The abstracts and titles of all 239 references were examined to determine if they met the five inclusion criteria:

- Subjects are Galliformes.
- Study reports at least one of the following: amount of dustbathing (or vertical wing shakes), foraging in, pecking in, or time spent on substrates. Substrate use for nesting purposes is not within the scope of this meta-analysis.
- Because many countries are moving toward non-cage systems (35), and because substrate has a larger presence in non-cage systems, tested substrates must be practical for non-cage systems. In other words, they must be able to absorb moisture, so studies that assessed preference for astroturf, plastic or feed as a bedding were excluded.
- Study measured the amount of behavior performed as a percentage, count, or time spent. For example, measuring the time spent dustbathing on sand (time spent dustbathing on sand/total time dustbathing). Due to inability to synchronize all data types/units with the information provided, studies that only provided demand curves, or that measured the number of birds performing a behavior could not be used. For example, measuring the percent of birds dustbathing on sand (percent of birds dustbathing on sand/total number of birds).
- Experiment must be set up as a substrate preference test.

**Figure 1:**
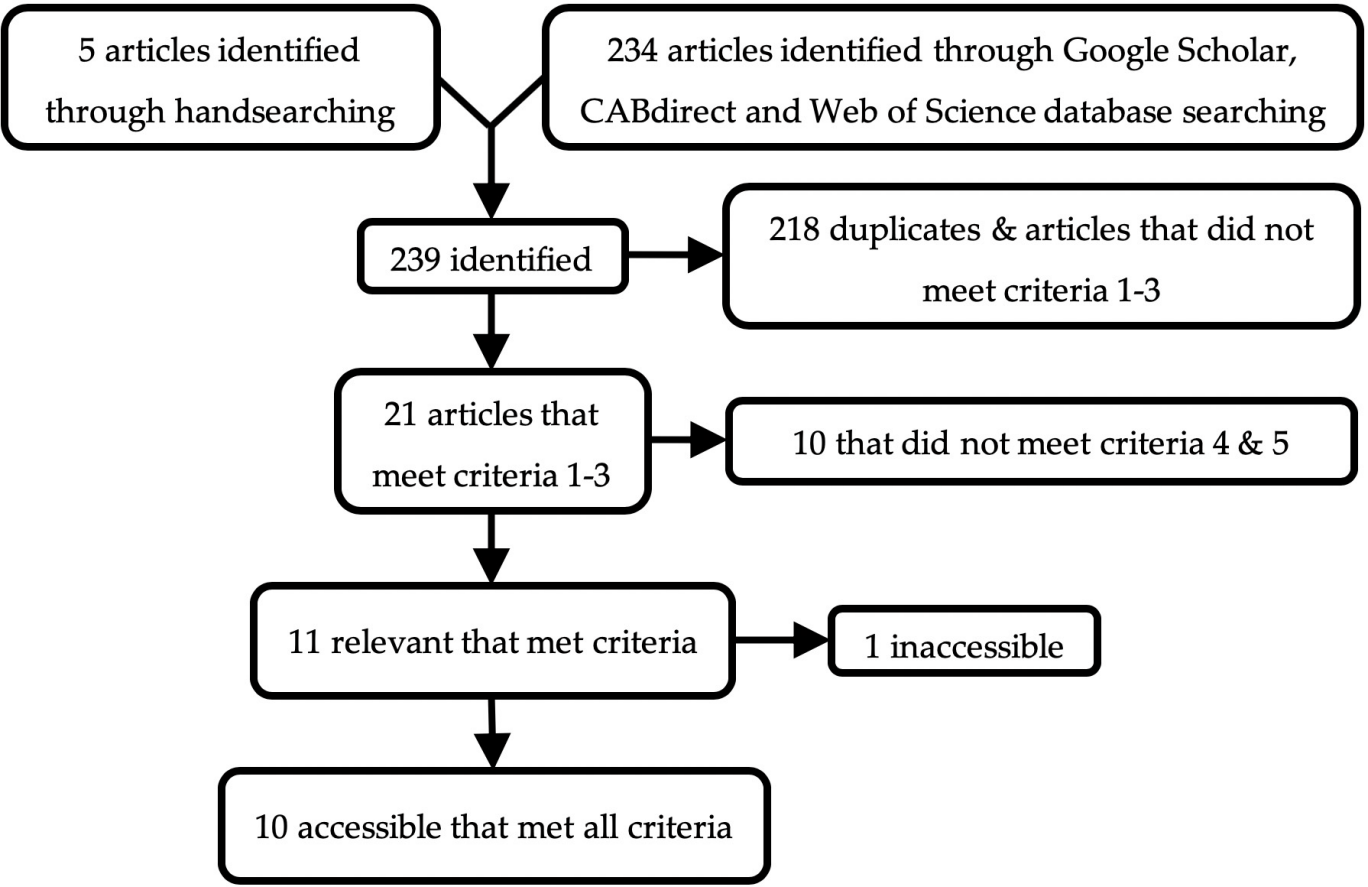
Schematic of the literature search.

Of the 239 articles found, ten were accessible and met these criteria (Figure 1). All accessible, relevant articles that met criteria used strains of chickens as subjects. One article described the effect of substrate on the behavior of Japanese quail (*Coturnix japonica*) (36); however, it was excluded as the study was not a preference test. Web Plot Digitizer software (37) was used to extract data from many of these articles that only reported outcomes through graphs. A summary of the database used for this meta-analysis is reported in Table 1. Due to the small number of studies that met the aforementioned criteria, and the diversity of substrates tested, all beddings excluding peat moss, sand and wood were grouped together as “other.” This category included a variety of beddings such as straw, paper, feathers, rice hulls, and wheat bran. Since only two studies reported foraging while many reported pecking and scratching (the two behavioral components of foraging), these last two behaviors were combined to represent foraging for all studies. Vertical wing shakes were grouped with dustbathing, as it was used to measure dustbathing in several studies.

Table 1Database summary.

**Table:** 

**Publication/Reference number**	**Study**	**Birds observed^a^**	**Species (breed)**	**Days on substrate^b^**	**Enclosure area (m^**2**^)^c^**	**Stocking density^d^**	**Animals/m^**2**^ /day^e^**	**Substrate area (m^**2**^)^f^**	**Substrate depth (cm)**
Jong et al., 2007 (38)	1	16	Layer	21.00	6.75	0.59	0.03	1.13–2.25	5.0
Sanotra et al., 1995 (39)	2	12	Layer	0.04	0.41	4.94	118.52	0.03–0.04	4.0
Shields et al., 2004 (40)	3	52	Broiler	4.00	2.31	0.87	0.22	0.37–0.82	10.0
Villagra et al., 2014 (41)	4	32	Broiler	0.05	4.00	2.00	41.14	1.00	15.0
Shields et al., 2005 (42)	5	60	Broiler	49.00	9.30	1.08	0.02	4.65	2.5
Toghyani et al., 2010 (43)	6	80	Broiler	42.00	5.76	3.47	0.08	1.44	2.0
van Liere et al., 1990 (44)	7	23	Layer	5.00	3.90	1.54	0.31	0.36	12.0
Hogan and Vestergaard, 1992 (45)	8	10	Jungle Fowl	0.02	0.48	4.17	171.43	0.12	2.0
Petherick and Duncan, 1989 (46)	9	48	Layer	0.02	5.76	1.04	50.00	0.13	4.0
Guinebretière et al., 2014 (33)	10	60	Layer	4.00	1.05	3.81	0.95	0.49	3.0

a *Total number of birds observed in each study (some studies picked focal birds)*.

b *Number of days birds were allowed access to tested substrates (in enclosure area). Small numbers represent studies that moved animals into a separate area to observe*.

c *The size of the total enclosure, encompassing all substrate areas, in m^2^*.

d *Animals per m^2^; Stocking density = animals per group (not shown; some studies used focal birds) ÷ enclosure area (m^2^)*.

e *Proxy for degree of substrate soiling (higher numbers indicate more soiling); animals/m^2^/day = stocking density ÷ days on substrate*.

f *The area (m^2^) occupied by one substrate within the enclosure*.

**Table:** 

**Study**	**Outcome measure^a^**	**Substrates tested**					**Outcomes reported**				
		**Peat moss**	**Sand**	**Wood**	**Other^b^**	**None**	**Time spent**	**Dustbathing**	**Foraging^c^**	**Pecking**	**Scratching^d^**
1	Count	✓	✓	✓		✓	✓	✓	✓		
2	Time budget		✓	✓	✓			✓		✓	✓
3	Count		✓		✓	✓	✓	✓		✓	
4	Time budget		✓	✓	✓		✓	✓		✓	✓
5	Time budget		✓	✓			✓	✓		✓	✓
6	Time budget		✓	✓	✓		✓	✓	✓		
7	Count		✓	✓				✓			
8	Percent		✓	✓				✓		✓	
9	Count	✓	✓	✓				✓		✓	✓
10	Count	✓	✓		✓			✓		✓	✓

a *How behaviors were recorded by the study. Count = behaviors per time period; Time budget = time spent doing behavior divided by total time spent on a substrate; Percent = total time spent doing a behavior divided by substrate*.

b *Other substrates include: straw, feathers, recycled paper, rice hulls, and wheat bran*.

c *Foraging = (percent of pecking + percent of scratching) ÷ 2*.

d *Scratching was not reported without pecking. Therefore, it was combined to make foraging, and is not analyzed alone*.

All outcomes were converted to percentages of behavior performed on each substrate (time based). Count data was converted as follows:

(1) $$\begin{array}{r} Percent\;of\;behaviour\;x\;on\;substrate\;y\\ =\frac{Count\;of\;behaviour\;x\;on\;substrate\;y}{Count\;of\;behaviour\;x\;on\;all\;substrates\;in\;study}\times \;100, \end{array}$$

Behavioral time budgets were often broken down as a division of time spent on substrate by behavior (e.g., resting on wood + dustbathing on wood + foraging on wood = 100% of behaviors on wood). To convert this time budget to a division of a single behavior by substrate (e.g., dustbathing on wood + dustbathing on sand + dustbathing on straw = 100% of dustbathing), we used the following equation:

(2) $$\begin{array}{r} Time\;doing\;behaviour\;x\;on\;substrate\;y\\ =\left(\frac{time\;doing\;behaviour\;x\;on\;substrate\;y}{time\;doing\;all\;behaviours\;on\;substrate\;y}\right)\\ \times \;time\;on\;substrate\;y, \end{array}$$

followed by an adapted version of Equation (1).

### Model Development

The methodology employed for this meta-analysis is described in St-Pierre (47) and Sauvant et al. (48, 49). Within this approach, study is treated as a random effect, and within-study patterns are examined while accounting for sources of between study variance (as the random study effect, or via inclusion of covariates). Data were analyzed using SAS Studio (SAS Inst. Inc., Cary, NC). Relationships between fixed effects were first examined using the Proc Reg MAX R procedure to assess the significance of continuous variables (stocking density, substrate area, enclosure area, number of days with access to substrate, animals/m^2^/day, substrate depth, and grain size) on the outcomes (time spent, dustbathing, foraging, and pecking at substrate). Categorical variables [species (breed), outcome measure (to ensure no effect of measurement)] were manually assessed for significance on outcomes one at a time with bedding type (which was always included regardless of significant outcomes) by running the generalized linear mixed model (GLIMMIX). This preliminary analysis was utilized to advise model development within a GLIMMIX procedure considering the random effect of study. A *P* < 0.05 was the criterion for statistical significance and for inclusion of fixed effects in the analysis, while tendencies are reported at 0.05 ≤ *P* ≤ 0.1. The incidence of dustbathing, foraging and pecking on substrate followed a gaussian distribution whereas time spent on substrate followed a lognormal distribution. The cook's D influence analysis was then used with a mixed model to identify influential outliers in the gaussian models, while extreme observations exceeding ±3.4 (identified by Proc Univariate) were used to identify outliers in the lognormal model. However, no outliers were removed. The percentage of dustbathing, foraging, and pecking at substrate is shown as LS means ± SEM, and time spent is shown as omega backtransformed LS means ± SEM. Wald-type F-statistics were used to test general linear hypotheses, while differences for LS means were tested using *t*-tests. In our description of results, F_v1_,_v2_ denotes a Wald-type F-statistic on v1 numerator and v2 denominator degrees of freedom, and t_v_ denotes a t-statistic on v error degrees of freedom.

### Model Evaluation

Model evaluation was performed to assess model goodness of fit. Models were evaluated based on the normality of residuals, which were examined graphically and using the Shapiro-Wilk statistic. Normality and homogeneity of the random effect was observed graphically with QQ and sample distribution plots, and time spent, the only non-normal model, was evaluated for over dispersion by ensuring that ChiSquare/DF <1.0.

Further model evaluations were performed using the study-adjusted Y predicted values. These evaluations employed the use of percent root mean square prediction error (rMSPE%), which estimates the overall prediction error and identifies the best model as one with a smaller rMSPE% value. The mean square prediction error (MSPE) is calculated as:

(3) $$\begin{array}{r} \overset{n}{\underset{i=1}{\sum }}\frac{{\left(O_{i}-P_{i}\right)}^{2}}{n}, \end{array}$$

where *n* is the total number of observations, *O*_*i*_ is the observed value, and *P*_*i*_ is the predicted value. The MSPE was then decomposed to determine the model's mean bias (ECT), regression slope deviation (ER) and error from disturbance (random error; ED), as well as the percent of error that each of these make up (50).

Lastly, the model was evaluated using the concordance correlation coefficient (CCC), which identifies a model's relationship as perfectly unrelated (−1), not related (0) or perfectly related (1). The CCC is calculated as:

(4) $$\begin{array}{r} CCC=\;{R\;\times \;C}_{b}, \end{array}$$

where *R*—the Pearson correlation coefficient—measures precision and *C*_*b*_ measures accuracy. Precise models display means that are strongly associated to one another, while accurate models have predicted means that are close to observed means. Calculating *C*_*b*_ also requires two other variables: *v* and μ. A measure of scale shift, *v* expresses the change in standard deviation between predicted and observed values. Greater variance in the predicted data compared to observed is shown in a *v* value larger than one, while values less than one indicate a smaller variance. μ measures location shift relative to the scale, with negative μ values implying underprediction and positive values indicating overprediction (51).

## Results

### Results of Final Models

#### Percent of Time Spent

**Figure 2:**
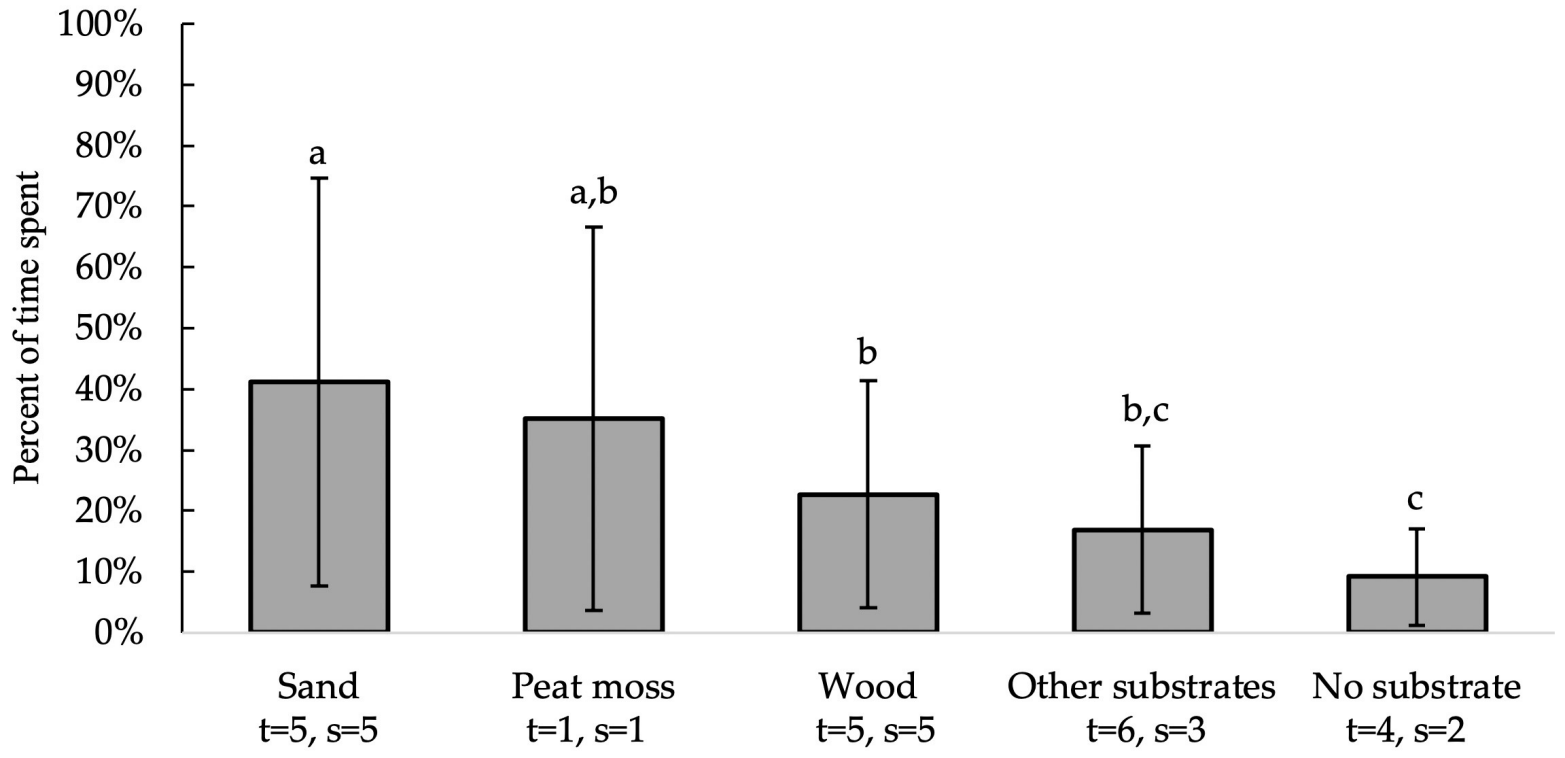
Percent of time spent (LS means ± SEM) on substrates (sand, peat moss, wood, other substrates and no substrate). Different letters indicate significant differences (*P* < 0.05). Number of treatments (t) and studies (s) that include each substrate are noted below each bar. The numbers of t and s differ because some studies used multiple floor substrates of the same kind with distinct differences (e.g., wire floors and a concrete “home” area for no substrate).

All independent variables were analyzed for significance in predicting percent of time spent on a substrate. Table 2 presents a summary of variables' effects on time spent. Bedding type [*F*_(4, 10)_ = 11.82, *P* = 0.0008], substrate area [*F*_(1, 10)_ = 39.18, *P* < 0.0001], and enclosure area [*F*_(1, 10)_ = 8.89, *P* = 0.0138] all significantly affected which substrate chickens preferred spending their time on Figure 2. Birds spent more time on sand (41 ± 33.6%) than on wood [23 ± 18.6%; *t*_(10)_ = 3.47, *P* = 0.0376], other substrates [17 ± 13.8%; *t*_(10)_ = 5.09, *P* = 0.0033], and no substrate [9 ± 8.0%; *t*_(10)_ = 5.58, *P* = 0.0017]. Additionally, birds spent more time on peat moss (35 ± 31.5%) than on no substrate [*t*_(10)_ = 3.67, *P* = 0.0280]. However, there was no significant difference between the amount of time chickens spent on peat moss, wood and other substrates (*p* > 0.05). Table 3 shows the predictive equations produced for time spent on floor substrates if all substrates are given at the same time.

**Table 2:** The significance of independent variables on outcomes.

**Independent variables**	**Models**			
	**Time spent**	**Dustbathing**	**Foraging**	**Pecking**
Bedding type	*	*	*	*
Substrate area	*			
Enclosure area	*			
Number of days birds were allowed access to tested substrates				
Stocking density				
Animals/m^2^/day				
Species (breed)				
Substrate depth				
Grain size				

Green indicates a significant effect (P < 0.05), yellow indicates a tendency (0.05 ≤ P ≤ 0.1), and red indicates a non-significant effect (P > 0.1). Starred (*) variables were included in model equations.

**Table 3:** Equations for time spent.

**Percent of time spent on:**	**Equation^a^**
Sand	= 1.3 (±0.17) + 2.0 (±0.32) × Substrate area (m^2^) – 1.0 (±0.34) × Enclosure area (m^2^)
Peat moss	= 1.1 (±0.33) + 2.0 (±0.32) × Substrate area (m^2^) – 1.0 (±0.34) × Enclosure area (m^2^)
Wood	= 0.7 (±1.84) + 2.0 (±0.32) × Substrate area (m^2^) – 1.0 (±0.34) × Enclosure area (m^2^)
Other substrates	= 0.4 (±0.18) + 2.0 (±0.32) × Substrate area (m^2^) – 1.0 (±0.34) × Enclosure area (m^2^)
No substrate	= −0.2 (±0.27) + 2.0 (±0.32) × Substrate area (m^2^) – 1.0 (±0.34) × Enclosure area (m^2^)

*Each equation represents the predicted time that chickens would spend on each resource if given all five options. The estimate and SEM are presented for each independent variable in the equation*.

a *The output of all equations must be backtransformed from a lognormal distribution to obtain the result on the original data scale (e^equation^)*.

#### Percent of Dustbathing

**Figure 3:**
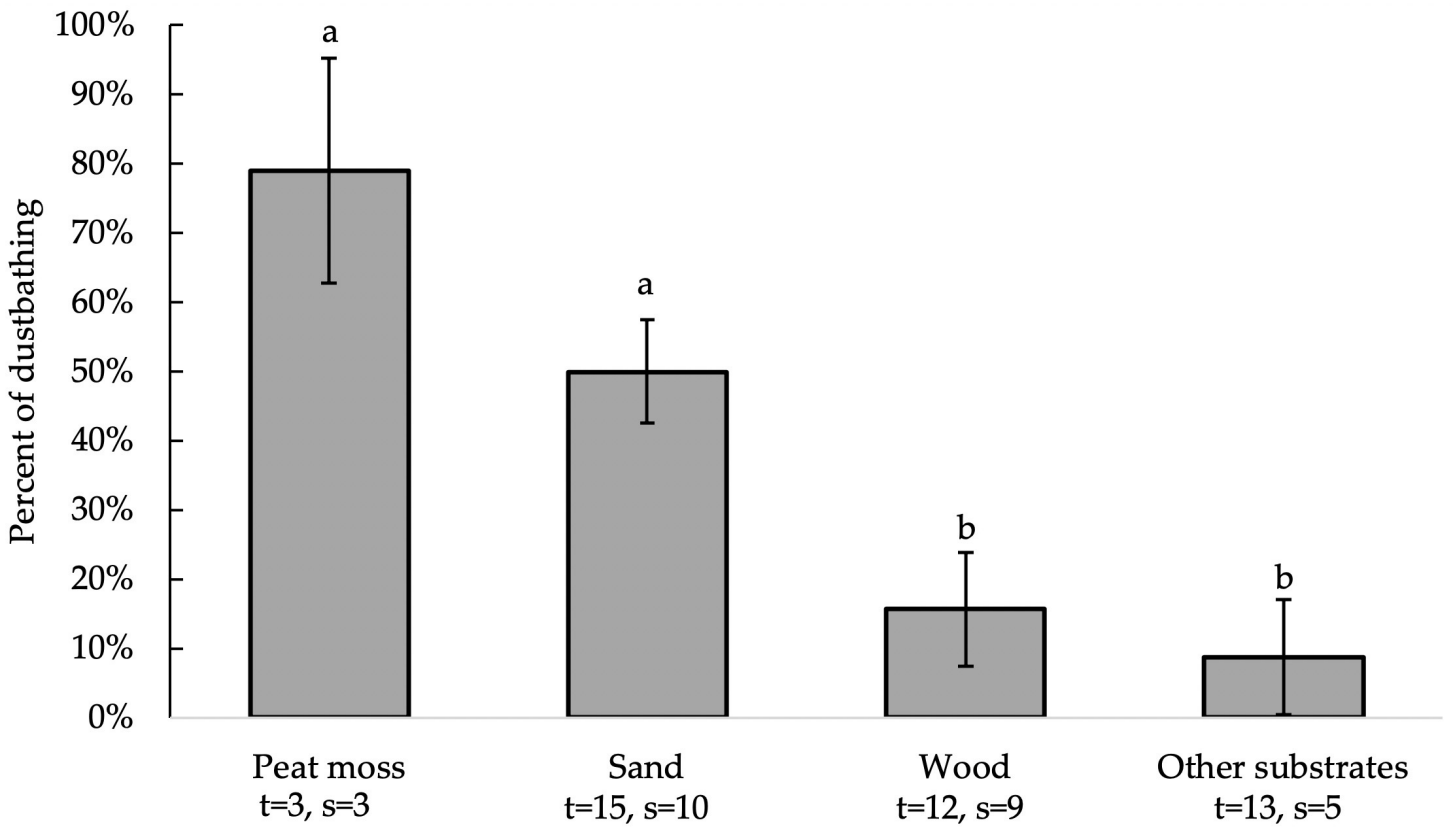
Percent of dustbathing (LS means ± SEM) on substrates (sand, peat moss, wood, and other). Different letters indicate significant differences (*P* < 0.05). Number of treatments (t) and studies (s) that include each substrate are noted below each bar. The numbers of t and s differ for sand and wood because some studies used multiple beddings of the same kind with distinct differences (e.g., different colors or grain sizes).

All independent variables were analyzed for significance in predicting percent of dustbathing. Table 2 presents a summary of variables' effects on dustbathing. Bedding type [*F*_(3, 30)_= 8.98, *P* = 0.0002] alone significantly affected substrate preference for dustbathing (Figure 3). Chickens dustbathed significantly more on peat moss (79 ± 16.3%) than on wood [16 ± 8.3%; *t*_(30)_ = 3.51, *P* = 0.0074] and other substrates (9 ± 8.3%; *t*_(30)_ = 3.87, *P* = 0.0029]. Chickens dustbathed more on sand (50 ± 7.5%) than on wood [*t*_(30)_ = 3.21, *P* = 0.0158] and other substrates [*t*_(30)_ = 3.85, *P* = 0.0031]. Percent of dustbathing was similar between peat moss and sand. Because bedding is the only variable that impacts the percent of dustbathing, the equations produced by this model are identical to the LS means ± SEM presented in Figure 3, and are therefore not shown.

#### Percent of Foraging

**Figure 4:**
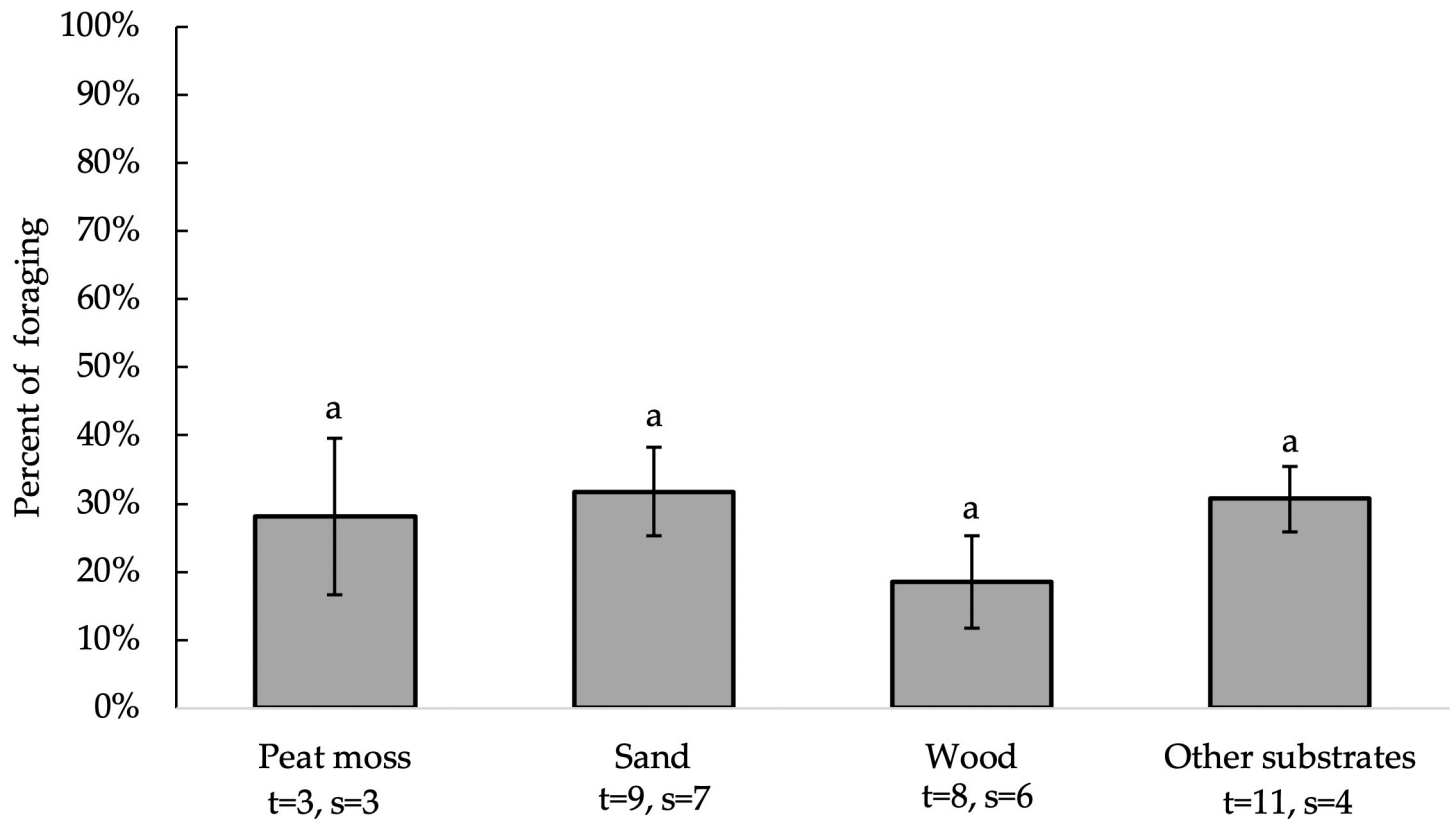
Percent of foraging (LS means ± SEM) on substrates (sand, peat moss, wood, and other). Different letters indicate significant differences (*P* < 0.05). Number of treatments (t) and studies (s) that include each substrate are noted below each bar. The numbers of t and s differ for sand and wood because some studies used multiple beddings of the same kind with distinct differences (e.g., different colors or grain sizes).

All independent variables were analyzed for significance in predicting percent of foraging. Table 2 presents a summary of variables' effects on foraging. None of the analyzed variables, including bedding type [*F*_(3, 21)_ = 80.73, *P* = 0.5430], significantly affected substrate preference for foraging behavior (Figure 4). Birds spent equal percentages of time foraging on peat moss, sand, wood and other substrates. Furthermore, analysis of the residuals vs. other factors revealed that none of the independent variables significantly affected the accuracy of model predictions (data not shown).

#### Percent of Pecking

**Figure 5:**
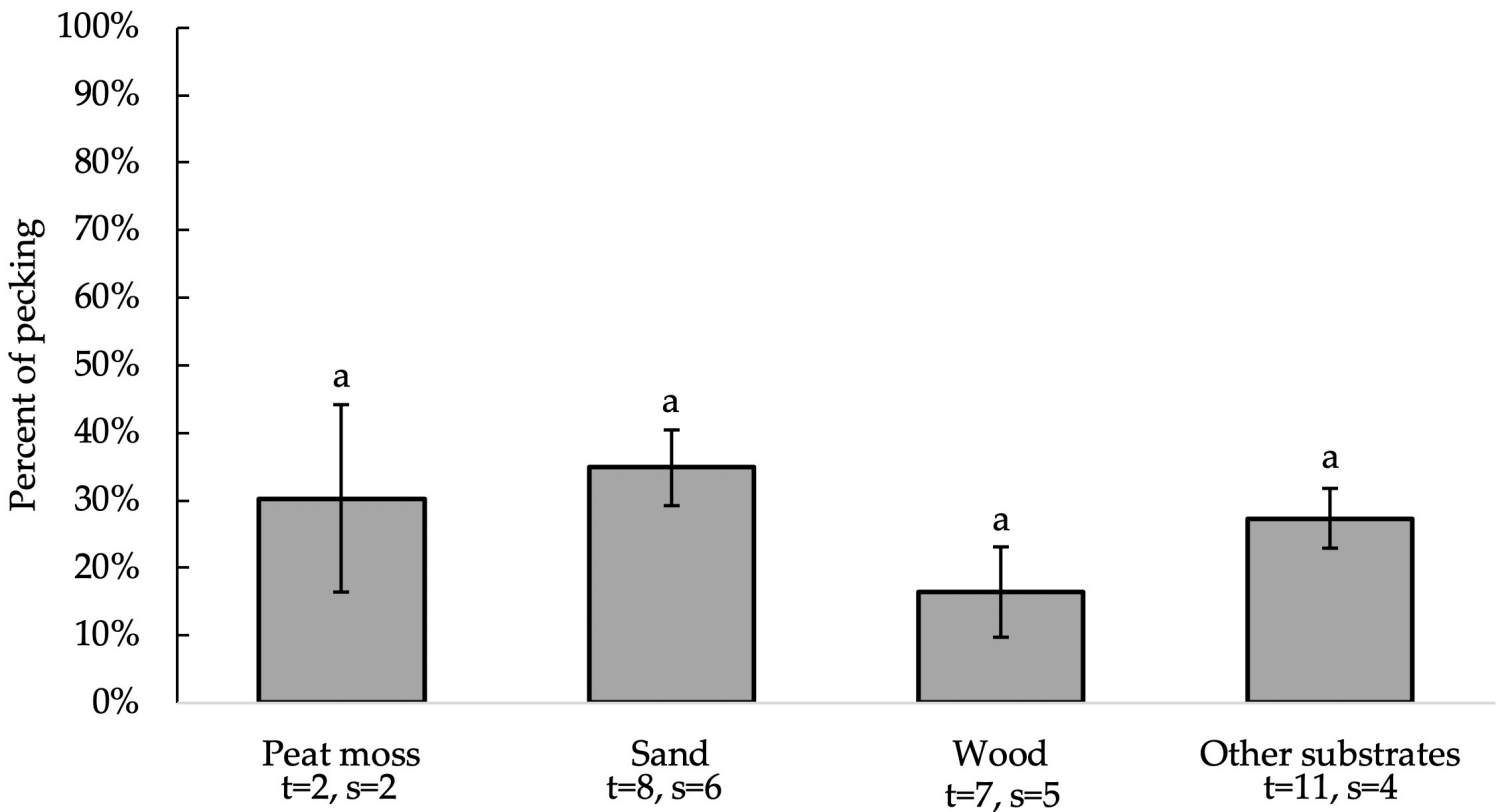
Percentage of pecking (LS means ± SEM) on substrates (sand, peat moss, wood, and other). Different letters indicate significant differences (*P* < 0.05). Number of treatments (t) and studies (s) that include each substrate are noted below each bar. The numbers of t and s differ for sand and wood because some studies used multiple beddings of the same kind with distinct differences (e.g., different colors or grain sizes).

**Figure 6:**
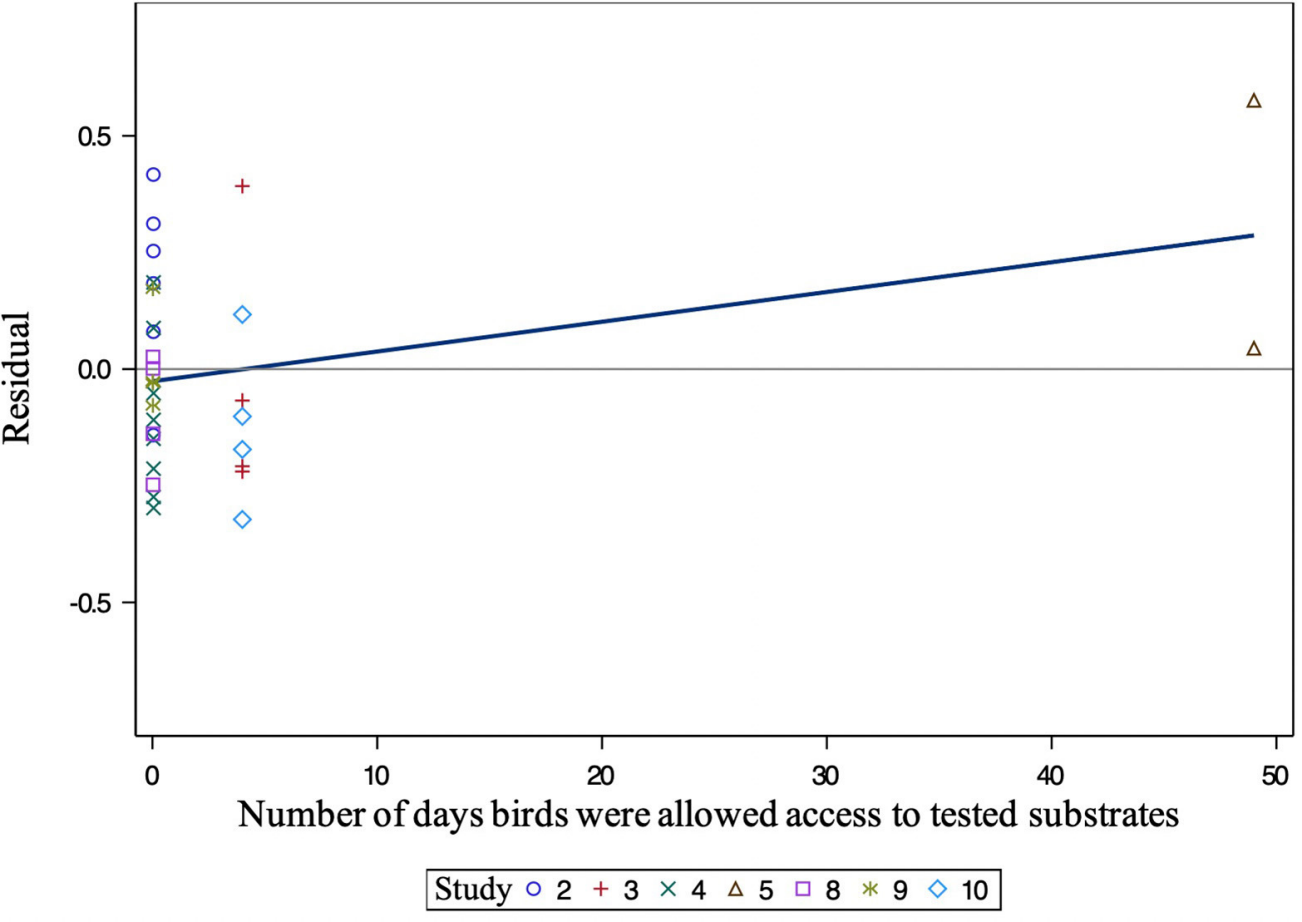
Residual of pecking vs. the number of days birds were allowed access to tested substrates. Different symbols indicate different studies. All numbers correspond to a study (Table 1).

All independent variables were analyzed for significance in predicting percent of pecking. Table 2 presents a summary of variables' effects on pecking. Similar to foraging, none of the analyzed variables, including bedding type [*F*_(3, 22)_ = 1.30, *P* = 0.3002], significantly affected substrate preference for pecking behavior at substrate (Figure 5). However, the number of days birds were allowed access to each tested substrate impacted the percentage of pecking (0.05 ≤ *P* ≤ 0.1). Further analysis of the residual vs. other factors revealed similar results to foraging. However, plotting the residual vs. the number of days birds were allowed access to each tested substrate (Figure 6) showed a non-constant distribution of residuals across days on substrate. One study in particular (42) influenced the model to over-predict higher values, which decreased the pecking model's accuracy.

#### Model Evaluation

Model evaluation statistics based on the study-adjusted *Y* values for each of the final models are presented in Table 4. These results provide a metric of each model's ability to predict the identified outcome, and how well the model described the observed variation (be it due to fixed or random effects). Results indicate that, given its high CCC, the time spent model has both high accuracy (C_b_) and precision (R), describing the data well. However, the breakdown of rMSPE% shows that almost 10% of error is due to regression (ER%). Evaluation of the observed vs. adjusted predicted values shows that this error due to regression is driven entirely by observations of no substrate. Given the variety in what may be considered “no substrate” (e.g., wire flooring, a home compartment with feed and water etc.), this discrepancy between observed and predicted outcomes in the “no substrate” category makes sense.

**Table 4:** Final model evaluation parameters by outcome (time spent, dustbathing, foraging, pecking).

	**Time spent**	**Dustbathing**	**Foraging^a^**	**Pecking^b^**
rMSPE%^c^	21.1597	84.1658	73.9937	78.0356
ECT%^d^	0.0541	0.0000	0.0000	0.0000
ER%^e^	9.4228	0.3797	14.2886	14.5253
ED%^f^	90.5232	99.6203	85.7114	85.4747
CCC^g^	0.9410	0.6000	0.0168	0.0887
R^h^	0.9421	0.6686	0.0231	0.1085
C_b_	0.9988	0.8974	0.7272	0.8171

a, b *Non-significant equations*.

c *Root means squared prediction error, % of mean*.

d *Error due to mean bias; % of total MSPE*.

e *Error due to regression; % of total MSPE*.

f *Error due to disturbance; % of total MSPE*.

g *Concordance correlation coefficient; C = R × Cb*.

h *Pearson correlation coefficient; measure of precision*.

Conversely, the distribution of rMSPE% in dustbathing is mostly weighted onto random error. However, despite this expected, preferred distribution of error, the CCC of dustbathing is relatively low. This may be because, while the model shows good accuracy, it also has a relatively low precision (R), implying that a great deal of random error is yet to be accounted for. Similarly, the foraging and pecking models showed good accuracy but very low precision, which is unsurprising given that both of these models are non-significant.

## Discussion

The meta-analysis presented herein systematically reviewed chickens' preferences for substrates based on the time spent on a given substrate, as well as dustbathing, foraging, and pecking behaviors on the substrate. While the search criteria for the meta-analysis included Galliformes, the eligible studies, in the end, only included chickens (laying hens, broilers, or red jungle fowl) as test subjects. The effect of substrates on the behavior and preferences of other commercially farmed Galliformes such as pheasants, guinea fowl, Japanese quail, bobwhite quail, and turkeys is therefore unknown. Nevertheless, the stimulating and motivating qualities of substrates for dustbathing, pecking, and foraging is expected to be similar, if not the same, for other commercially relevant Galliformes (36, 52–54).

We found that bedding type, substrate area and the enclosure area influenced where chickens chose to spend their time. Birds spent more time on areas with sand than on those with wood, other substrates, or no substrate. Interestingly, the time spent on peat moss was similar to all other substrates tested, although they spent more time on peat moss than on no substrate. These findings suggest that chickens may decide where they spend time based on the comfort and feel of the substrate. For example, compared to peat moss and sand, many wood shavings contain chemical irritants (55, 56) in addition to being physically abrasive. Therefore, it is likely that, when provided with an alternative, birds avoided physically uncomfortable beddings like wood. This would be particularly relevant for heavier birds like broiler chickens and turkeys that have reduced perching ability (57) and spend much of their time sitting or lying down (58). It is, however, important to note that few studies have examined the impact of various substrates on comfort behaviors [e.g., preening, stretching, etc.; (42, 43)]. The time spent on a substrate also correlated positively with amount of space it covered, with chickens spending more time on substrates that covered larger areas (Table 2). Given that many chickens like to explore, the positive effect of a larger substrate area was likely counterbalanced when the enclosure areas were also large, as birds tended to spend less time on any given substrate with increasing enclosure space.

It is, however, important to note that only five studies reported the amount of time spent on each tested substrate. Moreover, model evaluation revealed that, despite the model's high accuracy and precision, it had a high error due to regression likely caused by observations of no substrate. As such, the equations produced from the percent of time spent model should be taken with caution. Additionally, even though only one study reported time spent with peat moss, it was analyzed as a separate substrate due to its reputation as a preferred bedding (33, 34, 59). Thus, conclusions for peat moss based on this analysis, while interesting, should also be approached with caution.

We report that chickens' dustbathing preferences were only influenced by the bedding type. Birds dustbathed on peat moss and sand significantly more than on wood and other substrates. Since peat moss and sand look and feel like dirt: the natural dustbathing substrate for Galliformes, the birds may find the naturalness of these beddings appealing. Galliformes are also known to prefer dustbathing in substrates with low lipid content (20, 60) that are easily distributed throughout the plumage (friable and of small grain size (30)). It follows then that they would choose beddings that readily absorb lipids from plumage (16, 44) and that are less prone to caking. However, only three studies tested dustbathing preference for peat moss, while 10 studies used sand, making conclusions about chickens' preference for peat moss less robust than the findings for sand.

Bedding type did not influence chickens' preference for foraging or pecking at substrates. Moreover, unlike dustbathing and time spent on a substrate, chickens did not show a substrate preference to forage or peck in. Given that foraging and pecking are frequently performed, exploratory behaviors (23–25), studies may require greater contrast in bedding type to see preference for foraging or pecking. For example, offering birds the choice between highly palatable, nutritive substrates compared to inorganic, non-nutritive substrates (30). The number of days birds could access each tested substrate tended to affect the frequency of substrate pecking (Figure 6). Increasing the number of days exposed to substrates caused the model to over-predict, decreasing the accuracy of the pecking model. This over-prediction was largely driven by one study (42) that exposed birds to the same substrate for 49 days, whereas other studies that reported pecking exposed birds to tested substrates for 5 days or less. Therefore, proper understanding of the effect of the number of days birds were allowed access to tested substrates requires that more research examine pecking behavior on substrate over longer periods of time.

Most studies did not explicitly state if litter was cleaned out or if fresh bedding was ever applied on top of litter, making the true number of days birds were allowed access to tested substrates an estimated variable. Nevertheless, the proxy variable for cleanliness, animals per m^2^ per day, did not significantly affect any of the behaviors analyzed. With regards to foraging and pecking behavior, chickens are known to forage in and consume excreta in the absence of substrates (61, 62), and are reported to consume 5–24% of their group's excreta even when housed on bedding (63). Therefore, substrates may become equally attractive foraging and pecking material as they soil. Moreover, soiled bedding degrades to a soil-like consistency over time, and a study by Moesta et al. (64) found that soiled, degraded wood shavings were more stimulating and adequate for dustbathing compared to fresh wood shavings. The data presented here, in addition to the literature, would therefore support the idea that degree of soiling has little impact on the behaviors quantified in this meta-analysis.

In general, substrate characteristics are under-studied or inconsistently reported in the current literature. For example, only three of the 10 studies in this meta-analysis reported grain size of select substrates. Of these studies, none reported grain size for all substrates included in the preference test and five grain sizes reported were ranges. While every study reported dustbathing or its various behavioral elements, none reported caking which severely impact behavioral activities, including dustbathing. Other measures that might influence dustbathing, such as moisture content, analogous measurements for degree of substrate friability, or lipid content were all lacking from the examined studies, and may have contributed to the low precision of the dustbathing model. Reporting measurable or observable substrate characteristics that may influence Galliformes' preferences can contribute to understanding reported observations, improve study reproducibility (65), and applicability across species. Despite chickens' preference for sand and peat moss, farmers are reticent to their use due to labor requirements, wear and tear on equipment, cost, accessibility and unsustainability. Therefore, knowledge of important substrate qualities for dustbathing, foraging, and other high priority behaviors (19) can aid in the search for practical, cost-effective, sustainable, healthy, and enriching substrates. For example, knowledge of chickens' preference for low lipid content substrates for dustbathing and nutritive substrates for foraging led Guinebretiere et al. (33) to test laying hens' preference for wheat bran (evaluated in this meta-analysis under “other substrates”) compared to peat moss and sand. Refocussing on substrate characteristics may also reduce the number of animals used for substrate preference research, thereby satisfying one of the “3 Rs (Reduce, Replace, Refine)” of non-human animal research (66). Specifically, evaluations of substrate characteristics instead of, or before, performing multiple preference tests with different species of Galliformes could be used in the place of many exploratory preference tests using new substrates.

Finally, this meta-analysis also highlighted the need for consistent methods of measurement. For instance, all studies measured dustbathing, but this behavior was measured using six different observations: vertical wing shakes per hour, percent of vertical wing shakes, mean dust-baths per hen per day, mean total dust-baths, dust-baths per hour, and percent of time (on substrate) spent dustbathing. This variation in measurement methods and the lack of reported SEM prevented weighting studies by SEM.

## Conclusions

As the first meta-analysis of chickens' floor substrate preferences, this review confirmed that chickens preferred to dustbathe and generally spend time on sand and peat moss over wood shavings, other substrates and no substrate. However, only 30% of the studies used in the analysis examined preference for peat moss, suggesting that the data related to peat moss are less robust than those of sand. We also report that the bedding type, enclosure area and substrate area affected the time that birds spent on the tested substrates. Interestingly, none of the examined variables affected foraging and pecking behavior.

We noted that few studies reported physical and chemical properties of substrates, which, in the future, can be used to develop and discover novel beddings. Moreover, variations in data reporting and the lack of reporting standard error of the mean limited analysis.

## Data Availability Statement

The raw data supporting the conclusions of this article will be made available by the authors, without undue reservation.

## Author Contributions

VM, JE, and AH-M conceived and designed the study. JE provided statistical analysis support. VM analyzed the data and wrote the main manuscript. All authors reviewed and approved the final manuscript.

## Conflict of Interest

The authors declare that the research was conducted in the absence of any commercial or financial relationships that could be construed as a potential conflict of interest.
